# Utilizing Petroleum Coke for Hydraulic Fracturing Flowback and Produced Water Treatment - Targeting Dissolved Organics and Iron Removal

**DOI:** 10.1021/acsomega.5c11615

**Published:** 2026-02-05

**Authors:** Xiaomeng Wang, Tingyong Xing, Behnam Namsechi, Pan Huang, Lin Yang, Chunqing Jiang, Hongbo Zeng, Mohamed Ali

**Affiliations:** † Natural Resources Canada, CanmetENERGY Devon, 1 Oil Patch Drive, Devon, Alberta T9G 1A8, Canada; ‡ Faculty of Engineering, Chemical and Materials Engineering Department, 3158 University of Alberta, 9211 116 St, Edmonton, Alberta T6G 2H5, Canada; § Natural Resources Canada, Geological Survey of Canada-Calgary, 3303 33 Street NW, Calgary, Alberta T2L 2A7, Canada

## Abstract

Responsible hydraulic fracturing is important for the future of Canada’s oil and gas sector. Hydraulic fracturing operations use significant amounts of water. The flowback and produced water from hydraulic fracturing operations contain toxic chemicals and is stored on site. Alberta Energy Regulator records hundreds of surface spills of flowback and produced water per year. Nevertheless, to promote water reuse during hydraulic fracturing operations and reduce costs, industry uses fit-for-purpose water treatment methods to treat the flowback and produced water, targeting suspended solids and dissolved solids. In addition, the recent interest in the lithium content in hydraulic fracturing flowback and produced water has stimulated direct lithium extraction technology development across Canada. However, most of these technologies require a certain degree of organic removal. Traditional water treatment technologies are costly, and as such, further development in low-cost water treatment techniques is crucial to treat and reuse flowback and produced water. In this study, we utilized waste materials (i.e., petroleum coke) from the oil sands industry for hydraulic fracturing flowback and produced water treatment by employing an in-house coke activation method. Flowback and produced water samples from different locations in the Western Canadian Sedimentary Basin were analyzed and compared using a suite of analytical techniques. Dissolved organics were analyzed by high-resolution mass spectrometry using nontargeted analysis methods. The organic and iron removal efficiencies of different activated carbon products were compared. Results indicate that the surface area of coke is the primary factor influencing its adsorption capacity for dissolved organics; however, it does not significantly impact the efficiency of iron removal. Overall, this study demonstrated the potential of using petroleum coke for treating flowback and produced water, laying grounds for development of low-cost treatment technologies.

## Graphical abstract

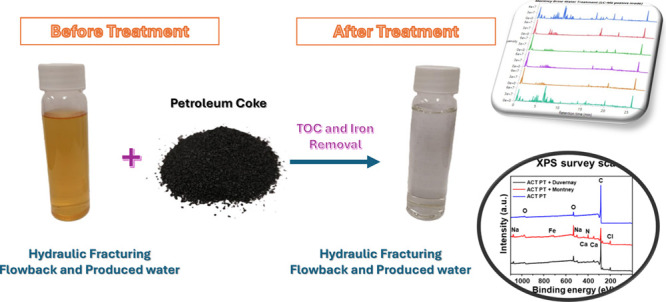

## Introduction

### Hydraulic Fracturing Operations in Canada and Beyond

The combination of horizontal drilling and multistage hydraulic fracturing (HF) is now widely used for the development of oil and gas reserves from low permeability reservoirs.^1^ Additionally, conventional wells that have run dry can be revived by horizontal drilling and HF to access previously unreachable oil reserves. Between 2000 and 2014, the production of shale and tight gas more than doubled in Canada, and by 2035, it is expected to represent 80% of Canada’s natural gas production.^2,3^ Globally, HF activities are concentrated in North America (US, Canada) and expanding in places like Argentina, China, and Russia.^4^ The overall rise in the adoption of HF has raised public concerns regarding the environmental implications of the practice, particularly its water use and the potential to contaminate groundwater.^5,6^ Therefore, environmentally sustainable HF is critical to the future of the oil and gas sector.

HF flowback and produced water (FPW) consists of a combination of the injected HF fluid and formation water originating from the target formation. HF fluid is a complex mixture of water and various chemical additives, while the formation water is often highly saline and contains a range of dissolved minerals and trace metals that naturally occur in the formation.^7^ The amount and chemistry of the HF FPW returned or produced from a well depend on many factors, including the geology, the reservoir condition, the fracture characteristics, the source of water used for fracking, the type of fracturing fluid, as well as the well completion practices.^8–10^ Due to its variable compositional complexity and high salinity, an understanding of HF FPW chemistry and toxicity is necessary to mitigate environmental contamination.^11^

In Canada, particularly in British Columbia and Alberta, all wastewater is collected and stored in enclosed tanks with secondary containment to avoid potential infiltration of slickwater or saline flowback water into the soil. No unlined surface ponds are currently being used in Canada.^6^ After fracking, the majority of this wastewater can be managed in one of two ways: injection in Class II disposal wells or treatment at a centralized facility for reuse or discharge.^10,12–14^ Injection in disposal wells is still the dominant methodology, but this adds significant cost to HF operations and increases the risk of HF FPW spills during transportation to injection sites. In 2015, 113 spills of HF FPW occurred in the Duvernay shale operations region alone.^11,15,10^ Clean up of HF FPW spills is challenging due to the variable and/or unknown chemical nature of FPW.^16^

For treatments, in current practice, the primary concern is the reduction of suspended solids, oil and grease, and scale-causing ions, but typically the dissolved organic matters are not considered.^17,18^ For example, the common methods for water reuse in Canadian HF operations would include bulk deoiling by three phase separators and skim tanks; neutralizing the pH of the water, followed by injection of hydrogen peroxide for disinfection and the oxidation of both H_2_S and iron. In addition, coagulant and flocculant are dosed, and the solids are then separated using floatation. Final deoiling is achieved using walnut shell filters. The process delivers about 15–100 mg/L oil-in-water concentrations for the treated water.[Fn fn1]

Removing dissolved organic matter prior to reuse is beneficial because high levels of organic matter can cause microbes to thrive and lead to microbial-induced corrosion in transportation lines, pumps, and drilling equipment.^14^ Further, high levels of organic matter resulted in gel-based fluids with lower peak viscosities,^19^ which is a critical characteristic for a fracturing fluid to carry and deliver the proppant.^20^ Thus, targeting organic matter removal is a key step in expanding future reuse management options that include treatment. It is reported that 31–44% of the world’s shale deposits are located in areas where oil or gas extraction could create or worsen water stress; 20% of shale deposits are in areas affected by groundwater depletion and 30% in irrigated land.^4^ Therefore, it is crucial to promote water reuse in HF operations.

Past study has demonstrated the ability of powdered activated carbon (PAC) to remove both polyethylene glycols (PEGs) and total petroleum hydrocarbons from HF wastewaters.^21^ As expected, an increased dose of PAC led to an improvement in the PEG removal for all waters tested. If the costs of PAC were potentially reduced through the use of petroleum coke, biochar, or other low-cost alternatives, it would be more plausible to incorporate PAC for the removal of dissolved organic matter.

### Petroleum Coke in Oil Sands

Some oil sand operators operate integrated oil sands surface mining and upgrading facilities in the Athabasca oil sands deposit in Alberta. To produce synthetic crude oil, fluid cokers were employed to reject carbon from the bitumen feedstock in the form of petroleum coke (PetCoke), which is a byproduct of the bitumen upgrading process. The carbon content of PetCoke typically exceeds 80 wt %.^22^ Approximately 15% of the extracted bitumen is converted to coke and stored on site.^23^ Alberta Energy Regulator estimated that on average, 31.3 kilotons of PetCoke are produced daily in the province of Alberta, Canada.^24^ Although high-quality PetCoke can be utilized for energy production or steel manufacturing, most are unsuitable for these applications due to high levels of sulfur, nitrogen, and heavy metals.^25^ As a result, by the end of 2024, approximately 164 megatons of stockpiled PetCoke waste have been accumulated in Alberta.^26,27^ Consequently, significant volumes of PetCoke are available for potential use, such as water treatment and reclamation.

Previous studies demonstrated that oil sands PetCoke could remove dissolved organic compounds from oil sands process water at both laboratory and pilot scales and thereafter produce treated water that was not acutely toxic toward rainbow trout and *Vibrio fischeri*.^28,29^ These results indicate that oil sands PetCoke could have potential applicability for the treatment of other oilfield wastewaters. As described earlier, HF is employed extensively in Canada and globally for the extraction of unconventional oil and gas resources, including shale gas and tight oil. Consequently, PetCoke generated in Alberta, Canada, could contribute to advancing “bitumen beyond combustion” by serving as a treatment material for FPW in a broader international context.

In this study, we utilized PetCoke from the oil sands industry for HF FPW treatment by employing an in-house thermal–chemical activation method. FPW samples from different locations in the Western Canadian Sedimentary Basin were characterized and compared using a suite of analytical techniques following the water treatment process. Interestingly, the results indicate that activated PetCoke removed not only dissolved organic compounds but also dissolved iron. Accordingly, the organic and iron removal performance of activated PetCoke was systematically compared with those of other PAC materials.

## Experimental Section

### Chemicals and Reagents

The PetCoke obtained from a bitumen upgrader was screened using an 18 mesh Tyler standard sieve (opening: 1 mm) to provide a uniform coke sample for this study. All screened coke was then dried at 150 °C for 24 h. KOH and HCl (both ACS grade, purchased from Fisher Scientific Canada) were used, respectively, for coke activation and product washing. In addition, three commercial activated carbons (named as AC1, AC2, and AC3) were obtained from an industry partner. Both PetCoke and commercial activated carbons were used to remove the dissolved organics from the FPW.

### Hydraulic Fracturing FPW Field Samples

In this study, FPW samples were taken from the surface tanks at HF operation sites in British Columbia and Alberta. Specifically, one sample was collected from a central processing pond receiving water from different wells that were hydraulically fractured in the Duvernay Formation in the Fox Creek area of Alberta. Similarly, the second sample was collected from another central processing pond in the Montney Formation in the Dawson Creek area of Northeast British Columbia.

### Petroleum Coke Activation

PetCoke was activated by a chemical process, leading to a several-fold increase in surface area and adsorption properties compared with raw PetCoke. Dry KOH powder was directly used, where a KOH/coke mixture with a mass ratio of 3:1 was manually ground in a ceramic mortar for 5 min. The mixture was transferred into an alumina combustion boat, which was then placed in a quartz tube furnace (Thermo Scientific, quartz tube: 70 × 6 cm) with nitrogen gas flow at a rate of 50 mL min^–1^ (as shown in Figure 1). Thereafter, the mixture was heated to the preactivation temperature (400 °C) at a rate of 10 °C min^–1^ and held for 0.5 h. Afterward, the mixture was heated to the activation temperature of 800 °C with the same ramping rate and dwelled for 1 h. In the tube furnace setup, water and KOH solution were used to remove toxic emissions such as CO and H_2_S (Figure 1). After thermal treatment, the product (activated PetCoke) was cooled to room temperature at a rate of 20 °C min^–1^ and subsequently neutralized with 1 M hydrochloric acid and deionized (DI) water until the filtrate reached a constant pH of 7. Finally, the activated PetCoke product was dried overnight at 150 °C.

**Figure 1:**
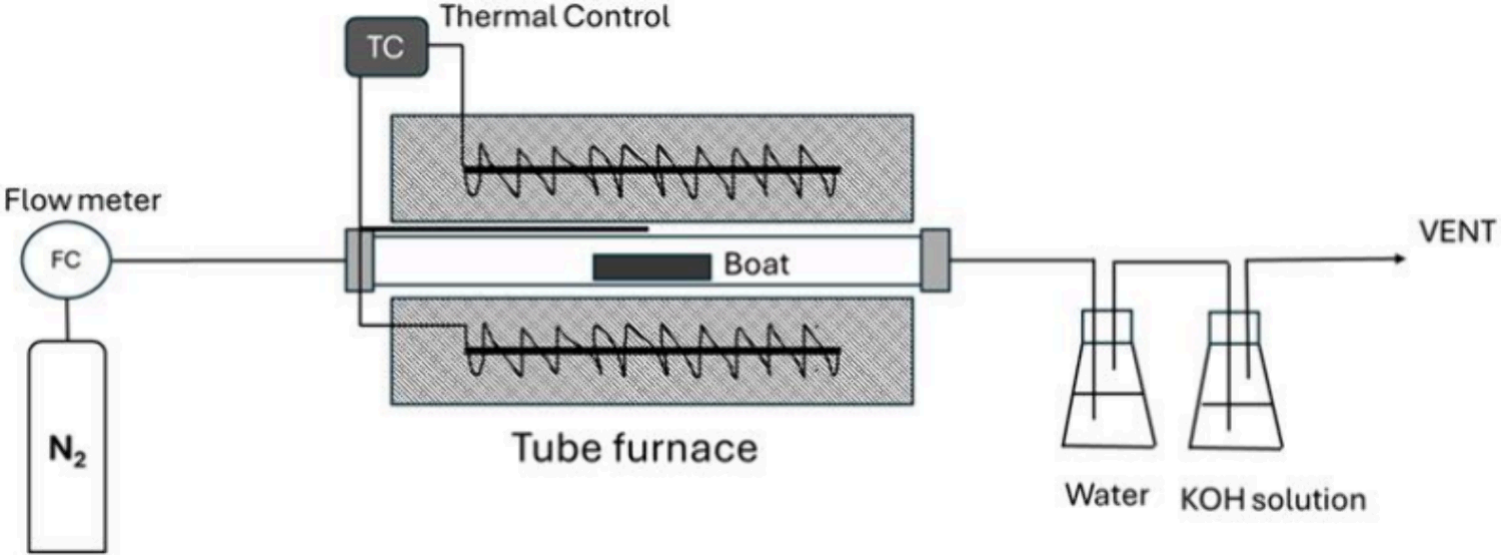
Schematic of the tube furnace setup used in the activation process for PetCoke.

### Bench Scale Adsorption Test

The adsorption test of dissolved organics was conducted using 60 mL amber glass jars at room temperature. Lab companion 5 plate stirrer was used for the study. First, 1 g of sorbents (commercial activated carbon or PetCoke samples) were added into each jar. Second, 60 mL of either DI H_2_O, Montney Brine, Duvernay Brine, or their diluted brines (at 1:1, 1:5, and 1:10) were added. The mixture was stirred at 600 rpm for 6 h and was allowed to settle overnight. The next day, the supernatant was taken using a pipet into sample vials for various water chemistry analysis. The adsorption isotherms were calculated by measuring the concentrations of dissolved organics before and after the adsorption tests in solution.

For the iron adsorption test, various concentrations of ferric chloride solution (at approximately 10, 30, 70, and 100 ppm of Fe) were made. 1 g of each commercial activated carbon, PetCoke, and activated PetCoke were used to adsorb the iron from 60 mL of ferric chloride solution. The other procedures are the same as those for the adsorption test of dissolved organics.

### Coke Characterization

#### Elemental Composition

The bulk composition (CHN/S) of PetCoke and activated carbon products were obtained using standard ASTM analytical methods (ASTM D5291/D1552). Oxygen content was determined by an in-house instrumental method utilizing an elemental oxygen analyzer.

#### Pore Volume and Surface Area Analysis

Pore volume and surface area properties were determined using a Micromeritics ASAP 2020 surface area analyzer by nitrogen adsorption isotherms using pure N_2_ gas (99.999%, Linde) at −196 °C. Before each adsorption measurement, approximately, 150 mg of sample was degassed under vacuum at 130 °C for 5 h to remove residual moisture or volatiles.^24^ The specific surface area of the sample was automatically determined from the adsorption isotherm by using the multipoint Brunauer–Emmett–Teller (BET) method. The pore size distribution was also obtained from the nitrogen adsorption isotherms using the density functional theory model for slit-shaped pores.^24^

### Water Characterization

FPW samples were characterized using various analytical techniques. Briefly, samples were filtered and then diluted to fit the calibration ranges of various analytical instruments used; the quantity and types of diluents used for sample dilutions were dependent on the analytical methods used for water chemistry analysis.

#### Inorganic Water Chemistry Analysis

Solution alkalinity, pH, and electrical conductivity were measured with a Man-Tech Associates PC-Titrate instrument equipped with a TitraSip module, which was calibrated using a Waters ERA P272-506 standard.

Chloride and sulfate concentrations were measured with a Thermo Fisher ICS 3000 ion chromatography (IC) system. Working standards of each analyte were prepared by using certified standards diluted with DI water. A commercial standard of 100 ppm of sulfate and 50 ppm of chloride was used as a quality control standard.

A Varian Vista-Pro 725 radial simultaneous inductively coupled plasma optical emission spectrometer (ICP-OES) equipped with a SPS3 autosampler was used to determine the concentrations of dissolved calcium, sodium, potassium, magnesium, sulfur, and trace metals. Working standards of each analyte were prepared using certified standard stock solutions.

#### Organic Water Chemistry Analysis

Soluble total organic carbon (TOC) was determined by using a Shimadzu TOC V-CPH instrument. The calibration standards used contained 0, 50, 100, 250, 500, and 1000 mg L^–1^ of carbon according to ASTM D7573. For the inorganic carbon analysis, sodium bicarbonate was used as a standard. For organic carbon analysis, potassium hydrogen phthalate was used as a standard.

To characterize detailed organic components of HF FPW, ultrahigh-performance liquid chromatography (UHPLC)-Orbitrap MS analysis was performed on solvent extracts at UVic-Genome BC Proteomics Centre, Victoria, Canada. Detailed procedures have been described previously.^30^ Briefly, water samples were extracted using dichloromethane following a similar procedure reported in the literature.^31^ Dried extract residue of each water sample was mixed with LC/MS-grade methanol. After vortex mixing, sonication, and centrifugation, UHPLC-high-resolution (HR) MS analysis was conducted for each sample by using a Thermo Ultimate 3000 UHPLC system coupled to a Thermo LTQ-Orbitrap Velos Pro mass spectrometer equipped with an electrospray ionization ion source. For compound detection, LC-MS runs were carried out in both positive-ion and negative-ion Fourier transform MS modes and in a mass range of *m*/*z* 70–2000. For LC separations, a Waters XBridge C18 (2.1 × 100 mm, 2.5 μm) column was used. 0.01% formic acid in water (solvent A) and 0.01% formic acid in acetonitrile/isopropanol (1:1, v/v; solvent B) were used as the binary mobile phase for gradient elution (i.e.: 0–1 min, 1% B; 1–15 min, 1–100% B; 15–17 min, 100% B) at a flow rate of 0.35 mL min^–1^ and a temperature of 40 °C. The acquired UHPLC-HRMS data sets were processed using the Compound Discoverer software from Thermo Scientific for peak detection, retention time shift correction, peak grouping, and molecular formula determination.

### Surface Characterization

Scanning electron microscopy (SEM) images and energy-dispersive X-ray spectroscopy (EDS) elemental mapping were obtained using a Hitachi S-4800 field-emission SEM instrument equipped with an Oxford Ultim Max EDS detector. Prior to characterization, the samples were mounted on aluminum stubs with conductive carbon tape and sputter-coated with a thin layer of Au using a Denton gold sputter coater. Room-temperature X-ray photoelectron spectroscopy (XPS) measurements were performed using a Kratos Axis (Ultra) spectrometer with monochromatized Al Kα (*h*υ = 1486.71 eV).

In addition, the zeta potential was measured by a Zetasizer Nano ZSP zeta potential analyzer (Malvern Panalytical Ltd., United Kingdom). All of the samples were ground into fine powders and tested in the simulated Montney and Duvernay brine solutions, which were made by adding salts in DI water at desired concentrations without dissolved organics.

## Results and Discussion

### Coke Properties and Characterization

In the literature, detailed economic evaluation of activated carbon production process from various raw materials (including pecan shells, bamboo, wood, used tires, PetCoke, carbon black, charcoal, and lignite) have been conducted.^32–35^ It is found that an attractive investment in activated carbon production requires selecting raw materials with a high product yield, adopting a chemical activation process, and pricing products based on their adsorption capacity for the target adsorbate.^32^ Calculations demonstrate that PetCoke meets all these criteria and represents the optimal feedstock for activated carbon production.^32^ Additionally, since PetCoke from the Alberta oil sands industry is a waste material, the raw material cost will be negligible and the return of investment will be even more significant compared to the other PetCoke resources.

In this study, we conducted detailed characterization of raw PetCoke, activated PetCoke, and commercial activated carbon materials. The properties of the raw oil sands PetCoke are given in Table 1. The sulfur content was 5.86 wt %; hence, the coke is considered as a type of high sulfur coke. As expected for a typical PetCoke, the raw coke sample had a limited pore volume and surface area.

**Table 1:** Elemental Analysis and BET Analysis

property, unit	raw PetCoke	activated PetCoke	commercial AC1	commercial AC2	commercial AC3
carbon, wt%	80.65	86.19	76.51	77.14	77.60
hydrogen, wt%	4.07	0.86	1.43	1.19	0.44
nitrogen, wt%	1.84	0.19	0.22	0.34	0.45
sulfur, wt%	5.86	0.17	0.40	0.57	0.58
oxygen, wt%	2.34	10.85	4.62	4.26	6.94
BET surface area, m^2^ g^–1^	0.54	2096.40	801.20	842.62	877.44
pore volume, cm^3^ g^–1^	<0.01	1.12	0.39	0.47	0.67
adsorption average pore width, Å	N/A	21.08	19.21	21.20	28.90

After activation, the sulfur content of the PetCoke dropped significantly due to the sulfur reacting with molten KOH to form volatile sulfur-containing gases during the activation process.^36^ Similarly, the contents of H and N were reduced in the activated coke products, albeit to a lesser extent. Interestingly, the oxygen content increased in the final activated coke products. It might be due to the fact that bitumen coke treatment with KOH affects the chemical and physical structures of the coke surface (i.e., functionalization of coke with C–O, C–O–C, and −OH motifs), resulting in an increased content of oxygen.^37,38^

The BET results of the coke samples are also listed in Table 1. The BET surface area and the total pore volume increased dramatically after activation, from below 1 to over 2000 m^2^ g^–1^ and from below 0.01 to over 1 cm^3^ g^–1^, respectively. The adsorption average pore width also increased to 21.08 Å after activation. We demonstrated in a previous study that the total pore volume consists of the micropore (pore size < 2 nm) and the large pore (pore size > 2 nm), and the micropore volume increased with the increase in the ratio of KOH/coke.^39^ Furthermore, the activation temperature had a great impact on the surface area and total pore volume for achieving better diffusion of KOH into bitumen coke, whereas the activation time and the preactivation temperature seemed to have negligible effect on both the surface area and total pore volume of the activated cokes.

For the three commercial activated carbon materials (i.e., AC1, AC2, and AC3), the surface area ranges from 800 to 880 m^2^ g^–1^, the pore volume ranges from 0.4 to 0.7 cm^3^ g^–1^, and the adsorption average pore width ranges from 19.21 to 28.90 Å. Both the surface area and pore volume were larger than those of the PetCoke sample before activation but smaller than those of the postactivation PetCoke. As the surface area and the pore volume are two critical factors for organic adsorption, the adsorption capacity for the activated PetCoke samples would likely be higher than those for the commercial products. In this study, adsorption isotherm tests on these carbon materials were conducted to investigate the relationship of the surface area, pore volume, and adsorption average pore width with the adsorption capacity of the carbon products. The results are presented in the following sections.

### General Water Chemistry of FPW

The general water chemistry data for HF FPW from both Duvernay and Montey operations are shown in Table 2. It appears that both water samples share a similar water chemistry profile, having high salinity and low pH. The only noticeable difference is the higher concentration of magnesium in the produced water from Montney formation than from the Duvernay formation, similar to previously published data.^30^ For trace metal analysis (Table 3), the Montney produced water sample exhibited a higher concentration of phosphorus (P), whereas the Duvernay produced water sample showed elevated levels of boron (B), silicon (Si), and barium (Ba). Also, it is worth noting that the concentrations of lithium in the produced water samples in both formations are around 60–80 ppm. Therefore, it is possible to recover this critical mineral from these wastewater samples if future lithium extraction technology development can achieve economical benefits.^40^

**Table 2:** General Water Chemistry for HF FPW

parameter	Duvernay formation	Montney formation
conductivity (mS cm^–1^)	204.3	209.0
pH	5.2	5.4
sodium (mg L^–1^)	67,820.8	66,550.8
magnesium (mg L^–1^)	1006.1	2203.8
sulfur (mg L^–1^)	179.5	220.3
chloride (mg L^–1^)	134,351	136,433
potassium (mg L^–1^)	<3800	<3800
calcium (mg L^–1^)	12,429.0	13,542.3
bicarbonate (mg L^–1^)	5.5	4.6
sulfate (mg L^–1^)	<1850	<1850
TOC (mg L^–1^)	529.8	609.3

**Table 3:** Trace Metals in HF FPW

	trace metals (μg L^–1^)	
	Duvernay	Montney
P	<96	7504
Sn	<116	<116
Tl	<112	<112
As	<140	<140
Se	<156	<156
Mo	<100	<100
Sb	<280	<280
Zn	1759	1434
Pb	<116	<116
Cd	<44	<44
Co	<56	<56
Ni	<20	<20
B	84,615	28,005
Si	27,730	17,046
Mn	5841	6046
Fe	23,954	28,356
Cr	<116	<116
V	<104	<104
Al	323	332
Be	<20	<20
Cu	<52	<52
Ag	<108	<108
Ti	<36	<36
Ce	<884	<884
Sr	1,231,480	1,265,240
Ba	20,671	12,740
Li	63,110	77,148

### Adsorption Capacity for Different Carbon Products

Adsorption is normally characterized by an adsorption isotherm, which is related to the free energy and entropy of the adsorption process, as well as to both the surface morphology and adsorption properties of the sorbents.^41–43^ The adsorption isotherm represents the equilibrium distribution between the concentration of species on the sorbent and the concentration in the aqueous phase at a constant temperature.^44^ For a wastewater treatment study, the extent of this adsorption determines whether it is feasible to utilize a particular adsorbent for the removal of organics from the water. When concentration changes are primarily governed by adsorption and the flow rate is low enough to achieve equilibrium, adsorption behavior can be described using models such as the Langmuir, Freundlich, Temkin, and D–R isotherms.^45,46^ Nevertheless, the adsorption of organic compounds at low concentrations onto sorbents can generally be described by a linear isotherm, a special case of the Freundlich model (a nonlinear isotherm eq 1):^47,48^

$$C_{s}=K_{d}C$$ 1

where *C*_s_ is the concentration of the solute on the sorbent (μg g^–1^), *C* is the concentration of the solute in the aqueous phase (mg L^–1^), and *K*_d_ is the equilibrium distribution coefficient or adsorption coefficient (mL g^–1^). The equation is valid provided that the density of the water is 1000 g L^–1^.

In this study, the concentrations of bulk dissolved organics in solution before and after the adsorption experiments were measured by TOC. Although the concentrations of individual organics in brine samples may be varied, the total concentrations of the dissolved organics were utilized for the adsorption isotherm calculations from a holistic approach perspective. Therefore, the *K*_d_ measured in this study was not the adsorption isotherm for each individual compound; rather, it was an analogue to the adsorption capacity of the sorbents, represented by different activated carbon products. The concentrations of TOC on the sorbent were calculated based on the difference between the two measured solution concentrations before and after each adsorption test. The adsorption coefficient *K*_d_ should be independent of the mass of the sorbent used when the concentrations of the chemicals in the system are low.^47,48^

Plots of *C*_s_ vs *C* for both Duvernay FPW and Montney FPW were constructed as shown in Figure 2. During the data analysis, the slope of the linear region of the curve was estimated as *K*_d_ for total dissolved organics reported in this study. Table 4 summarizes the *K*_d_ values for different sorbents. Results indicate that the activated PetCoke performs the best in terms of organic adsorption as it has the largest *K*_d_ values for both Duvernay FPW and Montney FPW. PetCoke before activation performs the worst as it exhibited the lowest *K*_d_ for both types of FPW. This result correlates well with the BET test and confirms that the sorbents with a large surface area and pore volume have higher adsorption capacity for organics. Nevertheless, even though the three commercial activated carbons have similar surface areas and pore volumes, their adsorption capacity varies for the same type of FPW (Table 4). The adsorption average pore width seems to have minimal impact on the adsorption capacity. These data indicate that other factors may play a role in terms of organic adsorption along with the surface area and pore volume. Furthermore, the sorbents tested in this study, with the exception of raw PetCoke, demonstrated higher adsorption capacity for the dissolved organics in Montney FPW than in Duvernay FPW. Considering that the amounts of TOCs in these two FPWs are similar (as shown in Table 2), it indicates that specific organic constituents in Montney FPW exhibited a greater tendency to adsorb onto the carbon surface, potentially due to their size, structure, or hydrophobic nature. As a result, a detailed surface analysis of the carbon materials and an organic analysis in the FPWs were conducted in this study to investigate this further.

**Figure 2:**
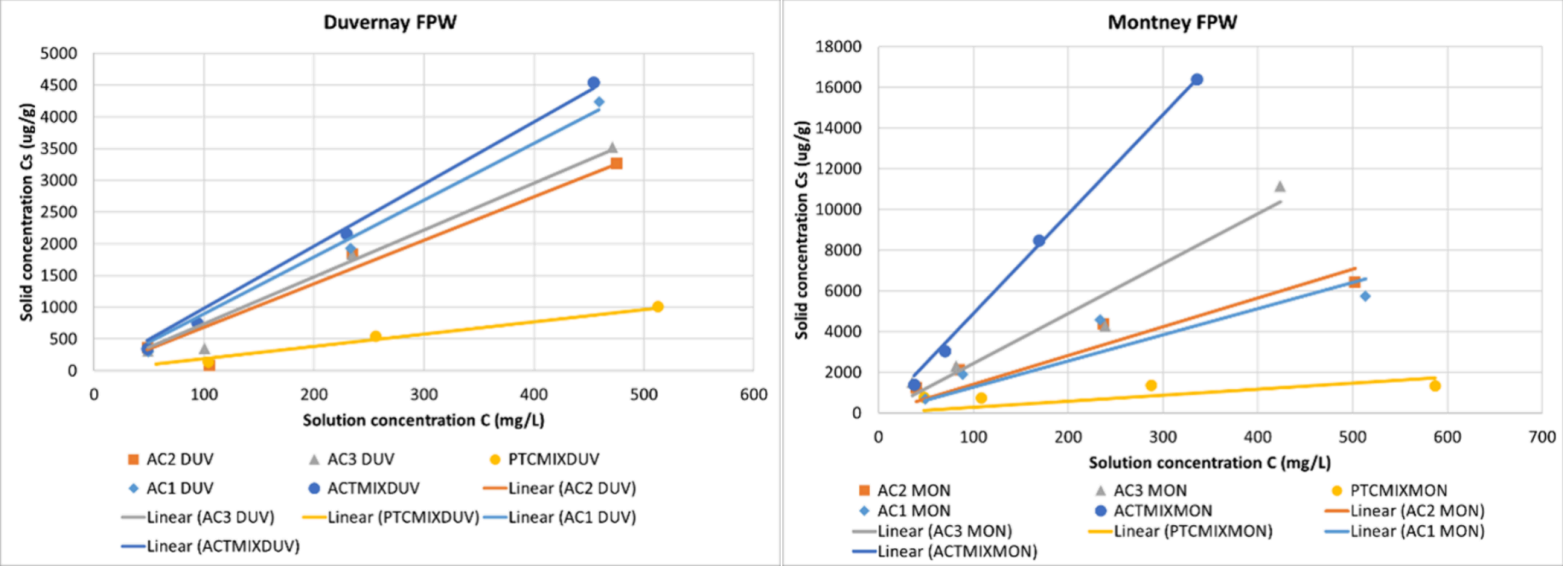
Adsorption capacity of dissolved organics in Duvernay FPW (left) and Montney FPW (right) for different carbon products. Activated PetCoke represented by dark blue dots; commercial AC1 represented by light blue diamonds; commercial AC2 represented by orange squares; commercial AC3 represented by gray triangles; and raw PetCoke before activation represented by yellow dots.

**Table 4:** *K*_d_ of Dissolved Organics in Duvernay and Montney FPWs for Different Carbon Products Used in This Study

*K*_d_ (mL g^–1^)	Duvernay FPW	coefficient of determination (*r* ^2^)	Montney FPW	coefficient of determination (*r* ^2^)
raw PetCoke	1.9	0.954	2.9	0.798
activated PetCoke	9.8	0.998	48.9	0.999
commercial AC1	9.0	0.997	12.8	0.935
commercial AC2	6.8	0.968	14.2	0.957
commercial AC3	7.4	0.989	24.5	0.976

### Sorbents Characterization before and after Adsorption

In this study, activated PetCoke samples were analyzed by XPS before and after the adsorption test for both Duvernay FPW and Montney FPW (Figure 3). Results suggest that the activated PetCoke sample before adsorption only shows the signals of C and O. In particular, C 1s spectrum is dominated by C–C (graphitic carbon, ∼284.5 eV) with smaller contributions from C–O–C/C–OH (∼286 eV) and minor C=O (∼287 eV).^49^ This is similar to typical activated carbon containing largely graphitic backbone with limited oxygenated defects.^50^ For the O 1s spectrum, it is broad but mainly with C=O (carbonyl/quinone) and some C–O (phenol/ether). As a result, XPS confirms that the activated PetCoke surface is mostly graphitic carbon with a modest amount of oxygenated functional groups (phenols, ethers, carbonyls). Limited N or metals are present.

**Figure 3:**
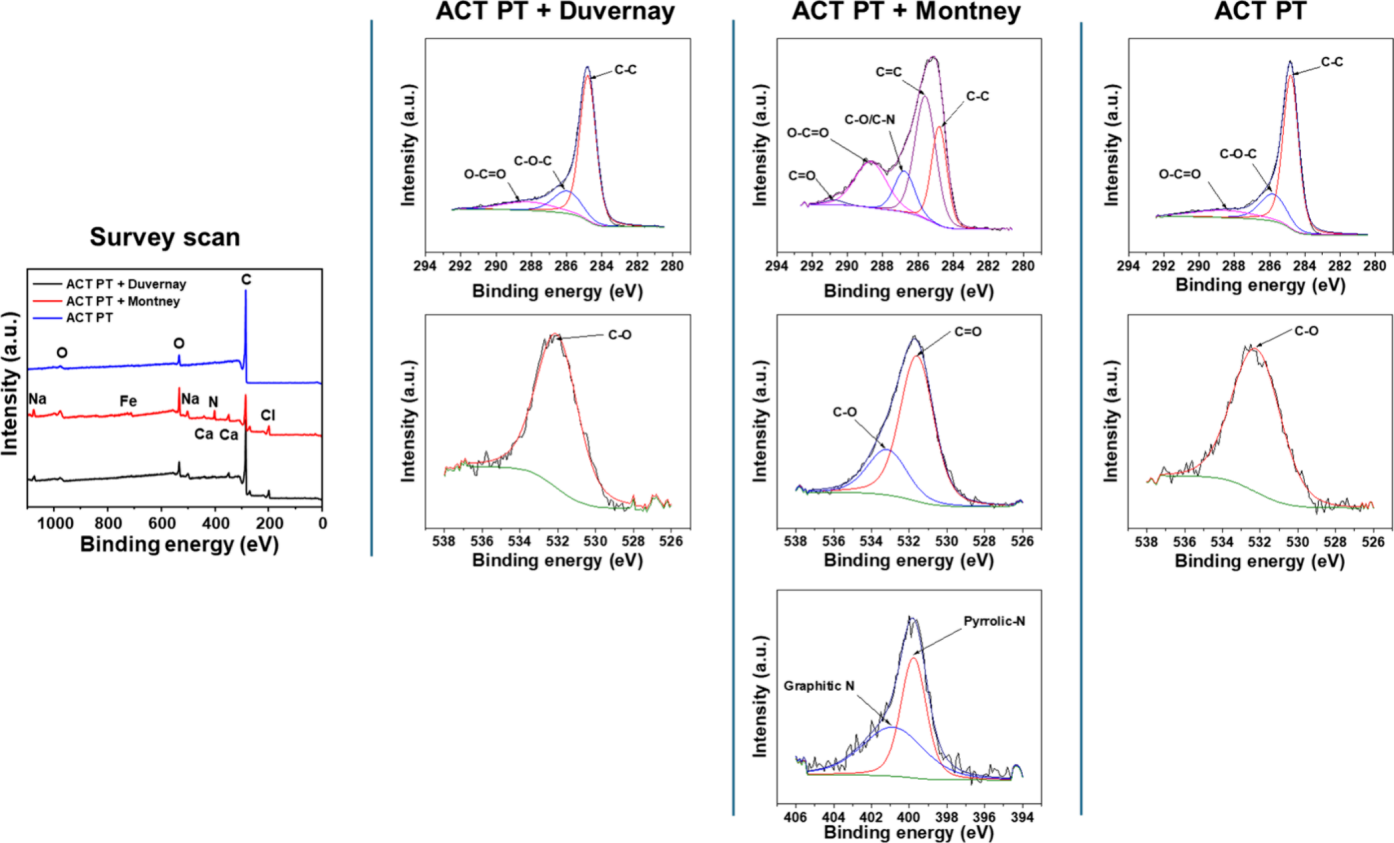
XPS results for activated PetCoke samples before and after water treatment. The “ACT PT” sample is the activated coke sample before water treatment. The “ACT PT + Duvernay” and “ACT PT + Montney” are the activated coke samples post water treatment in contact with Duvernay FPW and Montney FPW, respectively.

After contacting with Duvernay FPW, XPS of the activated PetCoke sample shows the signals of Na, Ca, and Cl. In particular, the C 1s spectrum shows evidence of the C–C backbone but also presents more pronounced oxygen functionalities contributed from C–O (alcohol/ether) and C=O (carbonyl/quinone). The O 1s spectrum demonstrates a clear C=O component (∼531–532 eV) plus broader C–O (∼533 eV).^49^ This suggests the adsorption of oxygenated organic or inorganic species from wastewater. The coke surface becomes enriched in oxygenated groups after adsorption. Inorganics (Na, Ca, and Cl) adsorb or precipitate on the surface. As the salt concentrations in the FPW are high, the Na, Ca, and Cl on the coke surface post adsorption are likely due to salt deposition.

For the activated PetCoke sample in contact with Montney FPW, its XPS spectrum shows the signals of N, Na, Ca, Cl, and Fe along with C and O. The C 1s spectrum shows multiple components, such as C–C (∼284.5 eV), C–O/C–N (∼286 eV), C=O (∼287 eV), and O–C=O (∼289 eV).^49^ This indicates both oxygen- and nitrogen-containing surface species. The O 1s spectrum contains C=O and C–O contributions, similar to the PetCoke in contact with Duvernay FPW, but has stronger signals. The N 1s spectrum contains two peaks, graphitic N (∼401 eV) and pyrrolic N (∼399 eV),^49^ which suggests the adsorption of nitrogen-containing compounds from Montney FPW. In addition, Fe detected in the XPS survey scan is likely due to the adsorption of Fe–O species or Fe complexed with surface oxygen/nitrogen groups. However, it is unclear if the Fe species adsorbed were inorganic compounds or organic complex since Fe 2p binding energies and satellite structures of many Fe species overlap.^51,52^ Nevertheless, this XPS spectrum confirms the adsorption of N- and Fe-containing species and inorganic ions. The carbon surface has more oxygenated groups and new nitrogen functionalities, which, in turn, confirms the higher adsorption capacity for the dissolved organics in Montney FPW.

In addition, SEM analysis was used to evaluate the surfaces and appearance of the samples in this study. Figure 4 shows the surface morphologies of activated PetCoke samples before and after water treatment. As shown in the SEM images of the activated carbon with various magnifications, a rugged and highly irregular surface is observed. Activated PetCoke has larger particles than PetCoke after water treatment. However, there seems to be no pore structures observed on the surface, which is likely due to the fact that nanopore structures could not be easily seen under regular SEM.^24^ In addition, activated PetCoke after contacting with Montney FPW demonstrates a rougher surface compared to both the before-treatment PetCoke sample and the PetCoke sample after contacting with Duvernay FPW.

**Figure 4:**
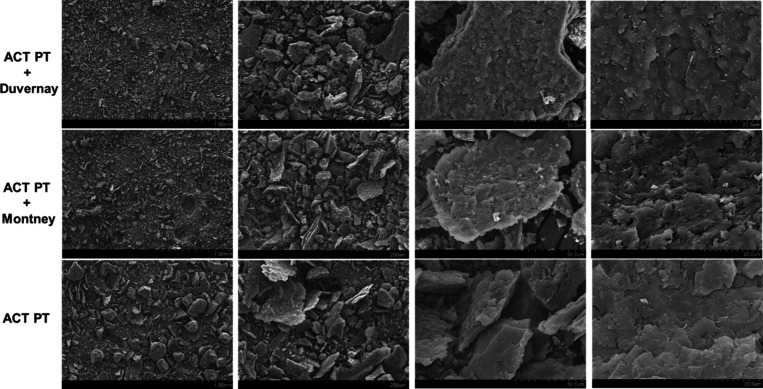
SEM images for activated PetCoke before and after water treatment. The “ACT PT” sample is the activated PetCoke sample before water treatment. The “ACT PT + Duvernay” and “ACT PT + Montney” are the activated PetCoke samples post water treatment for Duvernay FPW and Montney FPW, respectively. The scale bars for the SEM images are shown at the bottom right corner of each image; from left to right: 1 mm, 200 μm, 50 μm, and 20 μm.

SEM EDS was used to compare the surface compositions of the activated PetCoke before and after the wastewater treatment. The EDS results are presented in Figures S1–S3, indicating that the activated PetCoke contains 95.8% of carbon and 4.2% of oxygen; this is in deviation from the previous elemental analysis data [i.e., 86.2% carbon and 10.9% oxygen (Table 1)]. This discrepancy is attributed to the surface sensitivity and known quantification limits of EDS for light elements.^53^ After contact with FPWs, the oxygen content has increased, indicating that oxygen-containing functional groups have been adsorbed onto the surface. In addition, EDS results show salt precipitation along with organic adsorption. However, Fe adsorption has not been seen on EDS. Nevertheless, in consideration of the XPS results, it is confirmed that more organics have been adsorbed onto the surface of the activated coke after contacting the Montney FPW.

In addition, zeta potential measurements have been conducted on the coke samples including raw PetCoke, activated PetCoke, and activated PetCoke samples post water treatment. The zeta potential measures the electrical potential difference between the electric double layer of particles and the layer of dispersing agent around them at the slipping plane.^54^ Table S1 shows the zeta potential of various carbon samples in simulated brine solutions. PetCoke before activation has a negative zeta potential. After activation, its zeta potential becomes even more negative. It may be due to the fact that the surface of the activated carbon was occupied by electronegative groups such as oxygen-containing functional groups.^55^ The negative zeta potential generally enhances electrostatic repulsion between fine particles and, thus, promotes colloidal stability and homogeneous dispersion. Thus, small particles in suspension resist aggregation and tend to disperse homogeneously in the solution. Homogeneous dispersion of the adsorbent particle can obviously benefit from improving the adsorption efficiency of the adsorbent. After organic adsorption, the zeta potential of activated PetCoke becomes positive, indicating the adhesion of additional positively charged compounds onto the surface. This result correlates well with the XPS and EDS data.

### Dissolved Organic Removal

In this study, brine water samples before and after activated carbon adsorption were analyzed by LC-HRMS in both positive mode and negative mode. It is well-known that compounds (such as PEGs and organophosphates) that readily carry a positive charge will be detected by the positive-mode LC-MS spectra, whereas compounds (such as organic acids) that readily carry a negative charge will be detected by the negative mode LC-MS spectra. In addition, a greater number of peaks in the LC spectra indicate a wider variety of organic compounds, while the higher peak intensities reflect higher concentrations of these organics in the water. Since reverse-phase LC separation was used in this work, compounds eluted early normally have a lower mass-to-charge ratio and are more polar than the ones eluted later. As shown in Figures 5 and 6, Montney and Duvernay brine water before treatment have the largest number of peaks with the highest intensities compared to the other water samples after treatment for both positive-mode LC-MS and negative mode LC-MS. Commercial activated carbon treatment reduced the number of peaks in the raw brine water samples, but its performance was inferior to that of the activated PetCoke samples as the latter had the lowest number of peaks. PetCoke gave the worst treatment results, with the majority of the LC peaks remaining in the post-treatment water. For example, in the positive LC-MS analysis, a significant number of peaks with a retention time between 3 and 6 min, representing small polar compounds in Montney FPW, were removed by commercial activated carbon samples and activated PetCoke samples. However, these compounds were still present in the water sample after raw PetCoke treatment. Similarly, in the negative mode LC-HRMS analysis of Duvernay FPW, peaks with a retention time between 12 and 17 min disappeared after being treated by commercial activated carbon samples and activated PetCoke, but they were still present after raw PetCoke treatment. It should be noted that peak intensities in the positive-mode LC-MS spectra were much higher than those in the negative mode. Since most organics are more readily ionized under positive-mode conditions, higher intensities likely correspond to higher concentrations. Therefore, the positive-mode LC-MS results suggest that a greater portion of dissolved organics was removed from the Montney brine by both the activated carbon and activated PetCoke treatments, which corresponds well with the adsorption test results. This confirms that both the carbon materials and variations in the types of organics present in the brine samples influence the overall water treatment performance.

**Figure 5:**
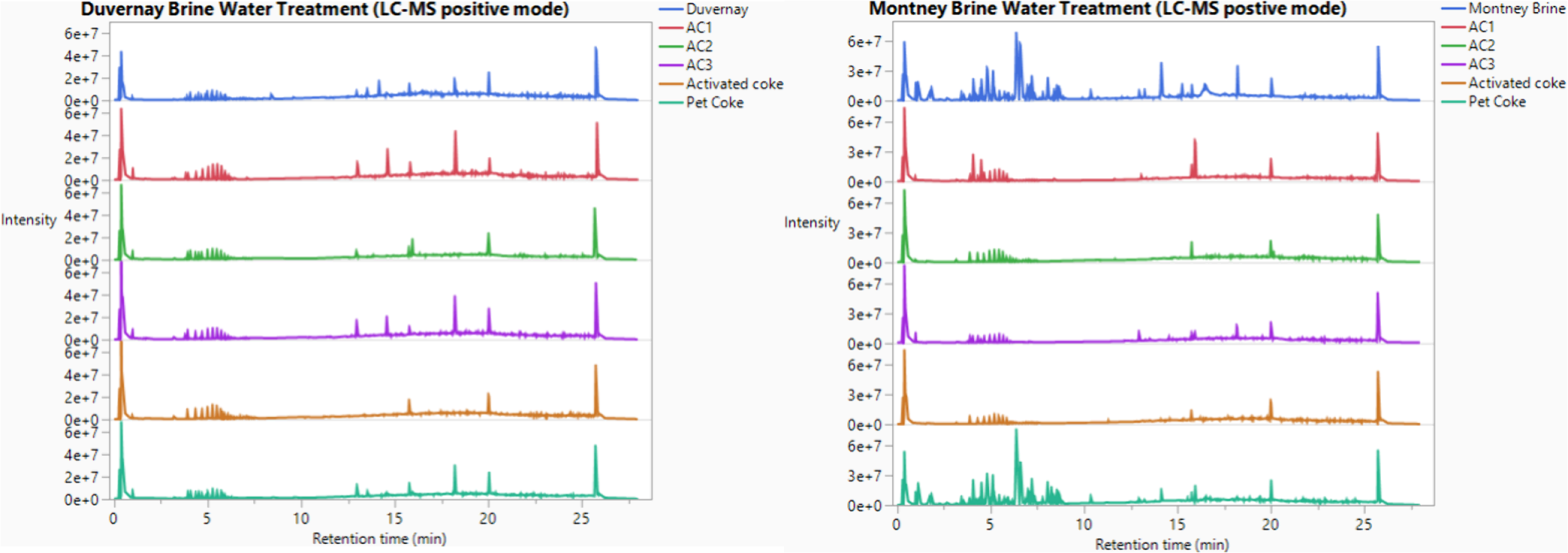
LC-MS positive-mode analysis for dissolved organics in Duvernay brine (left) and Montney brine (right) treated by using different carbon products. Raw water represented by top blue line; raw water treated by commercial AC1: red line, raw water treated by commercial AC2: green line, raw water treated by commercial AC3: purple line, raw water treated by activated coke: brown line, and raw water treated by PetCoke before activation: bottom dark green line.

**Figure 6:**
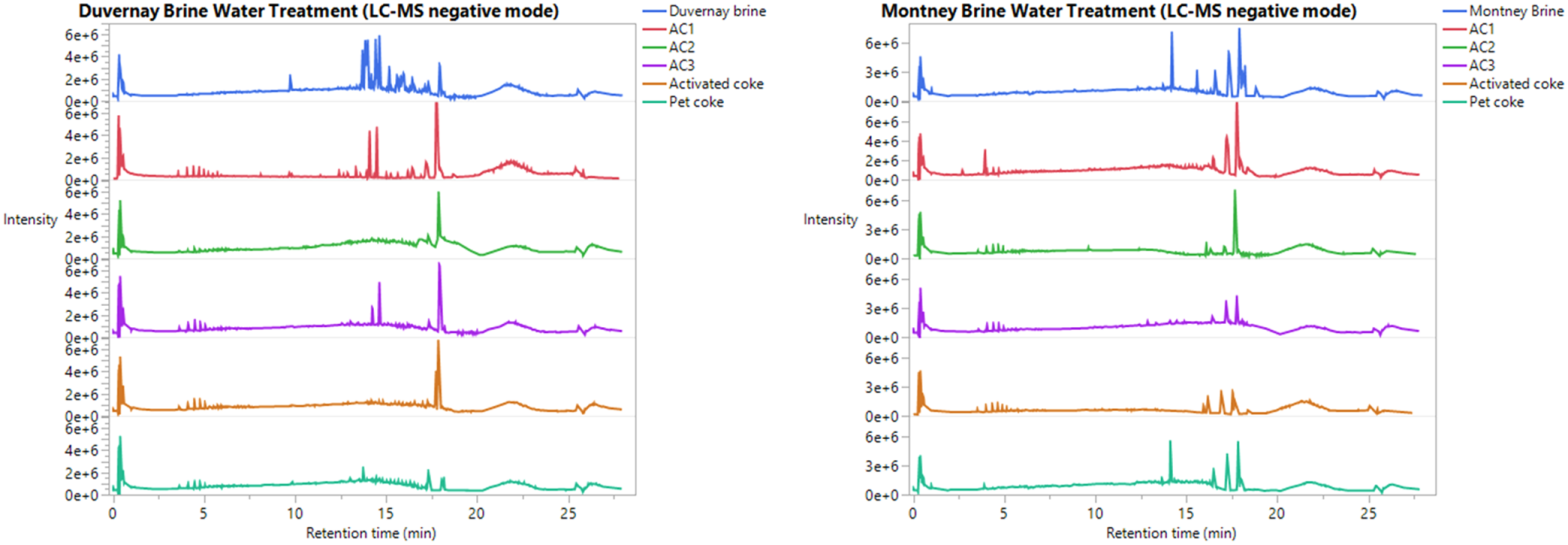
LC-MS negative mode analysis for dissolved organics in Duvernay brine (left) and Montney brine (right) treated using different carbon products. Raw water represented by top blue line; raw water treated by commercial AC1: red line, raw water treated by commercial AC2: green line, raw water treated by commercial AC3: purple line, raw water treated by activated coke: brown line, and raw water treated by PetCoke before activation: bottom dark green line.

Based on the fragmentation pattern given by tandem mass spectrometry, one could tentatively predict the type of compounds being removed by different carbon sorbents. As an example in this study, a detailed examination of the fragmentation patterns obtained from tandem mass spectrometry revealed the presence of potential aromatic amine compounds (C_14_H_15_N to C_17_H_21_N) in the Montney FPW, with retention times between 4 and 8 min. This group of compounds has been completely removed by commercial activated carbon and activated PetCoke sorbents. The fragmentation pattern for C_17_H_21_N is shown in Figure 7. As shown in the XPS results in Figure 3, graphitic and pyrolic N were predicted to be adsorbed on the activated PetCoke material surface. The results from tandem mass spectrometry correlates well with the XPS results. Further studies need to be conducted utilizing authentic standards to confirm the identity of this group of compounds.

**Figure 7:**
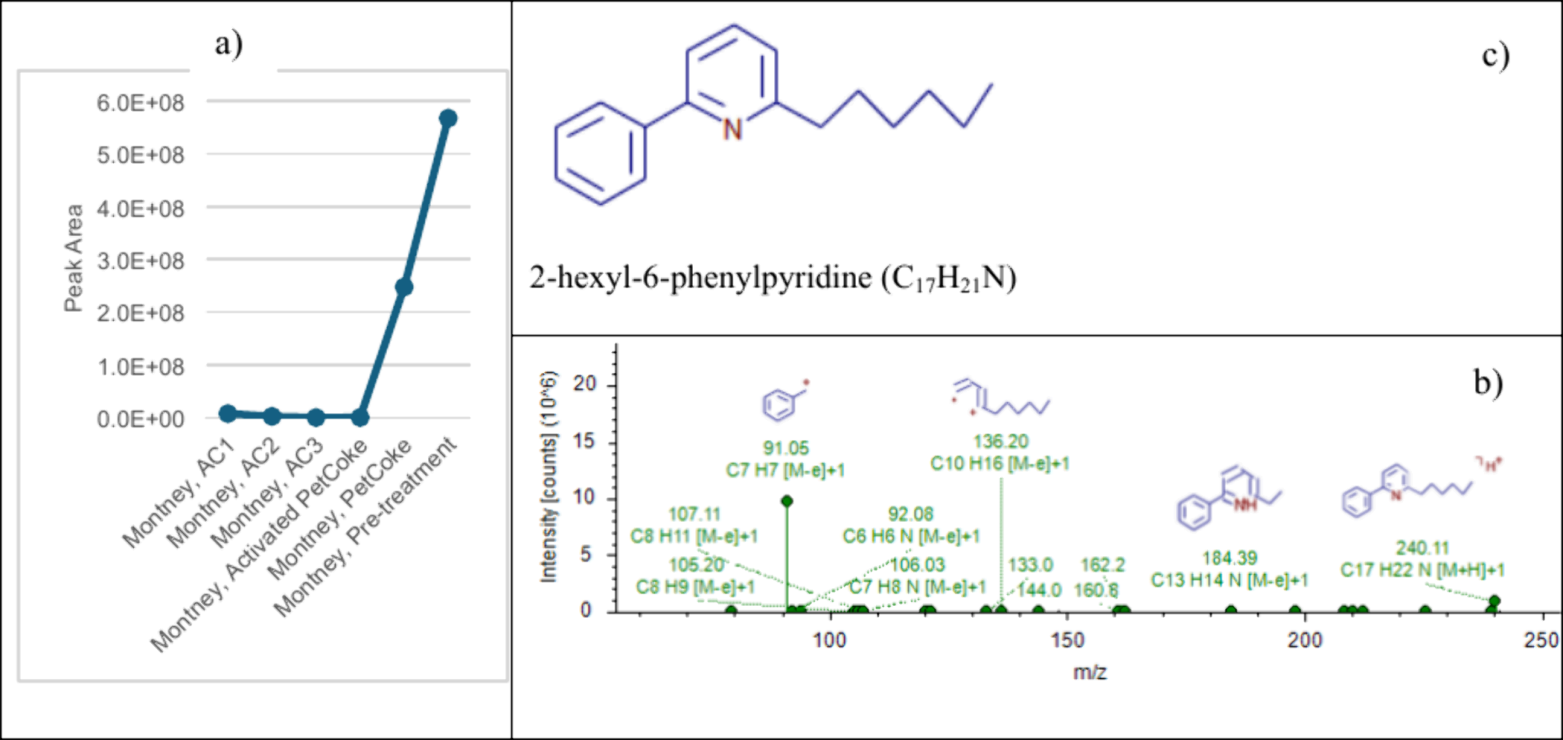
Peak area (a) and fragmentation pattern (b) for a suspected compound of interest [C_17_H_21_N shown in (c)] in Montney FPW under different carbon treatments; “Montney, AC1,” “Montney, AC2,” and “Montney, AC3” are the FPW treated by commercial carbon materials AC1, AC2, and AC3, respectively; “Montney, Activated PetCoke” and “Montney, PetCoke” are the FPW treated by activated PetCoke and raw PetCoke; “Montney, Pre-treatment” is the FPW before any treatment.

### Metal Adsorption

Trace metals in the FPW before and after the activated carbon treatment were compared. The results are presented in Table S2. Iron removal from FPW was observed for all activated carbon materials, whereas metals such as zinc were more effectively removed by activated PetCoke. As there is evidence of iron removal from the FPW by an adsorption test using different carbon materials, a systematic study was conducted for iron adsorption using various concentrations of ferric chloride solutions. The experimental procedure was the same as that for previous adsorption experiments using FPWs. The results are displayed in Table 5.

**Table 5:** Iron Adsorption Results for Commercial Activated Carbon, PetCoke, and Activated PetCoke

adsorbents	amount of adsorbents (g)	adsorbed Fe (mg g^–1^)
AC1	1.0	5154.9
AC2	1.0	976.9
AC3	1.0	5161.7
raw PetCoke	1.0	394.8
activated PetCoke	1.0	3096.9

It appears that commercial activated carbon AC1 and AC3 performed the best in terms of iron removal where the adsorbed Fe exceeded 5000 mg g^–1^ of adsorbents. Activated PetCoke samples were the second-best performer for Fe adsorption, where the adsorbed Fe saturated at about 3000 mg g^–1^ of adsorbents. The lowest capacity of iron adsorption was observed for the raw PetCoke before activation, where the adsorbed Fe content was only 400 mg g^–1^ of adsorbents. These results confirmed the iron removal capability of activated PetCoke and commercial activated carbon products. In addition, as noted previously, the surface area of the activated PetCoke is the largest among all carbon materials tested in this study. But, the activated PetCoke adsorbed less amount of Fe compared to the commercial activated carbon AC1 and AC3. These results indicate that the surface area may have minimal or secondary impact on Fe adsorption. In addition, the adsorption average pore size seems to have a negligible effect on the iron adsorption as AC1 and AC3 have different pore widths (Table 1). Previous research has shown that the iron adsorption capacity of activated carbon is mainly due to the oxidation and precipitation of Fe.^56^ Some references also indicate the possible iron-organic complex formation which caused the iron removal in conjunction with the organic removal.^57^ Further tests will be needed in the future to investigate the fundamental mechanism of iron removal by activated carbon materials.

## Conclusions

In this study, we conducted in-house activation of PetCoke by heating under alkaline conditions and compared the in-house activated PetCoke with raw PetCoke and commercial activated carbons for removing dissolved organics from hydraulic fracking FPW. In addition, we have investigated the metal ion removal capabilities of these carbon materials.

The results demonstrated that the activated PetCoke samples have a larger surface area compared to commercial carbon samples and thus have larger adsorption capacities for dissolved organics. For the raw PetCoke before activation, it can also remove dissolved organics but with lower adsorption capacity compared with the other activated carbon materials tested in this study. Additionally, the iron adsorption capacity of the activated PetCoke and the selected commercial activated carbons was confirmed in this study. Results indicate that the iron removal capacity has a minimal relationship with the surface area of the activated carbon materials. Due to operational needs and environmental concerns, the removal of dissolved organics and iron may both be needed for hydraulic fracking FPW treatment in the field. Therefore, activated PetCoke offers mutual benefits by simultaneously addressing both issues.

## Supplementary Material



## Footnotes

1 Private communications with industrial operators.

## Notes

EDS images and data of activated PetCoke before and after water treatment; zeta potential measurement of PetCoke samples in simulated brine solutions; and trace metal analysis in FPW before and after activated carbon treatment (PDF)
