# Supplementary material for: Utilizing Petroleum Coke for Hydraulic Fracturing Flowback and Produced Water Treatment - Targeting Dissolved Organics and Iron Removal
Source: ACS Omega. 2026 Feb 5;11(6):10390–401. doi: 10.1021/acsomega.5c11615 (PMC12917625; doi:10.1021/acsomega.5c11615)
Supplement: Supplementary file 1 [file ao5c11615_si_001.pdf]

Supplementary Information for:

Utilizing petroleum coke for hydraulic fracturing flowback and produced water treatment-Targeting dissolved organics and iron removal

Xiaomeng Wang[1*], Tingyong Xing[1], Behnam Namsechi[1], Pan Huang[2], Lin Yang[2], Chunqing Jiang[3], Hongbo Zeng[2], and **Mohamed Ali[1]**

1. Natural Resources Canada, CanmetENERGY Devon, 1 Oil Patch Drive, Devon, Alberta T9G 1A8, Canada
2. University of Alberta, Faculty of Engineering, Chemical and Materials Engineering Department, 9211 116 St, Edmonton, Alberta T6G 2H5, Canada
3. Natural Resources Canada, Geological Survey of Canada-Calgary, 3303 33 Street NW, Calgary, AB T2L 2A7, Canada

*Corresponding author: xiaomeng.wang@nrcan-rncan.gc.ca

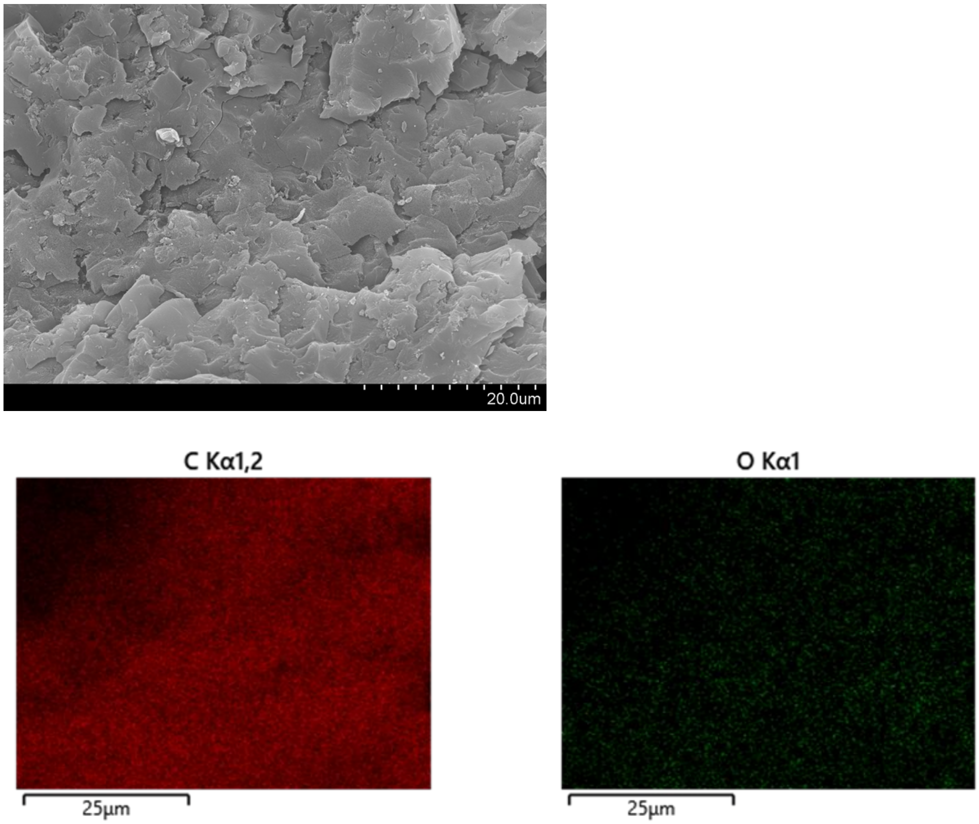

Element	Wt%
**C**	95.81
**O**	4.19

Figure S1 – EDS images and data of activated PetCoke before water treatment

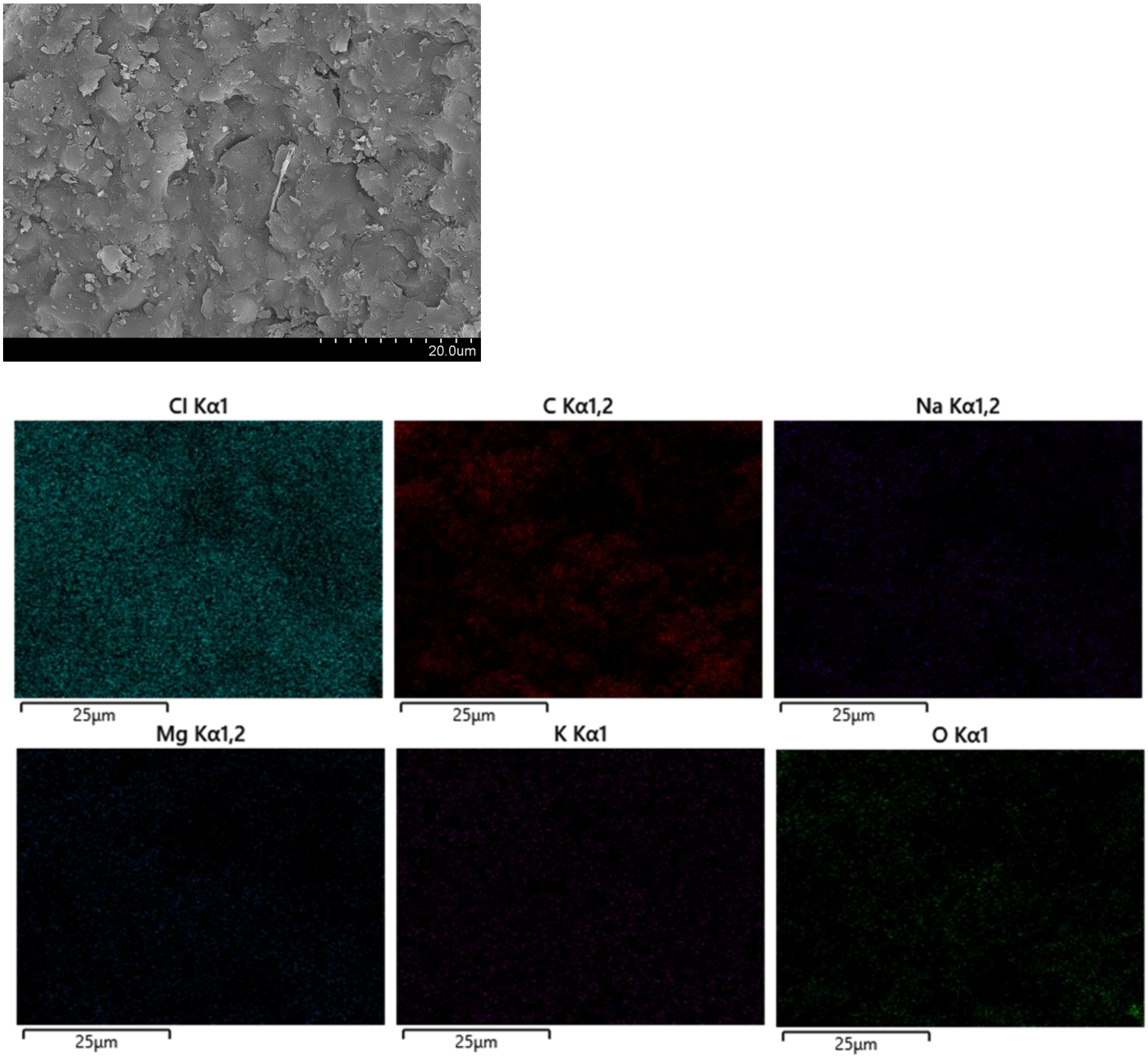

Element	Wt%
**C**	68.95
**O**	5.23
**Na**	0.74
**Mg**	0.27
**Cl**	16.48
**K**	0.63
**Ca**	7.71

Figure S2 – EDS images and data of activated PetCoke after contacting with Duvernay Brine

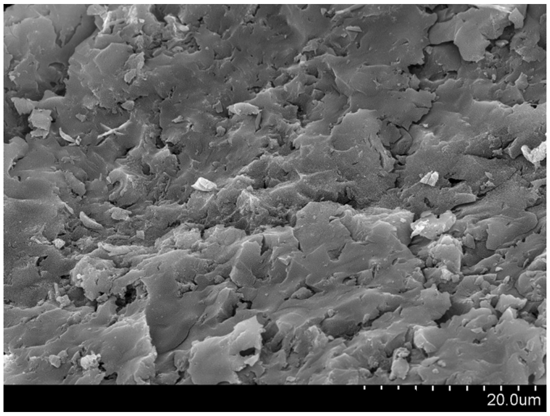

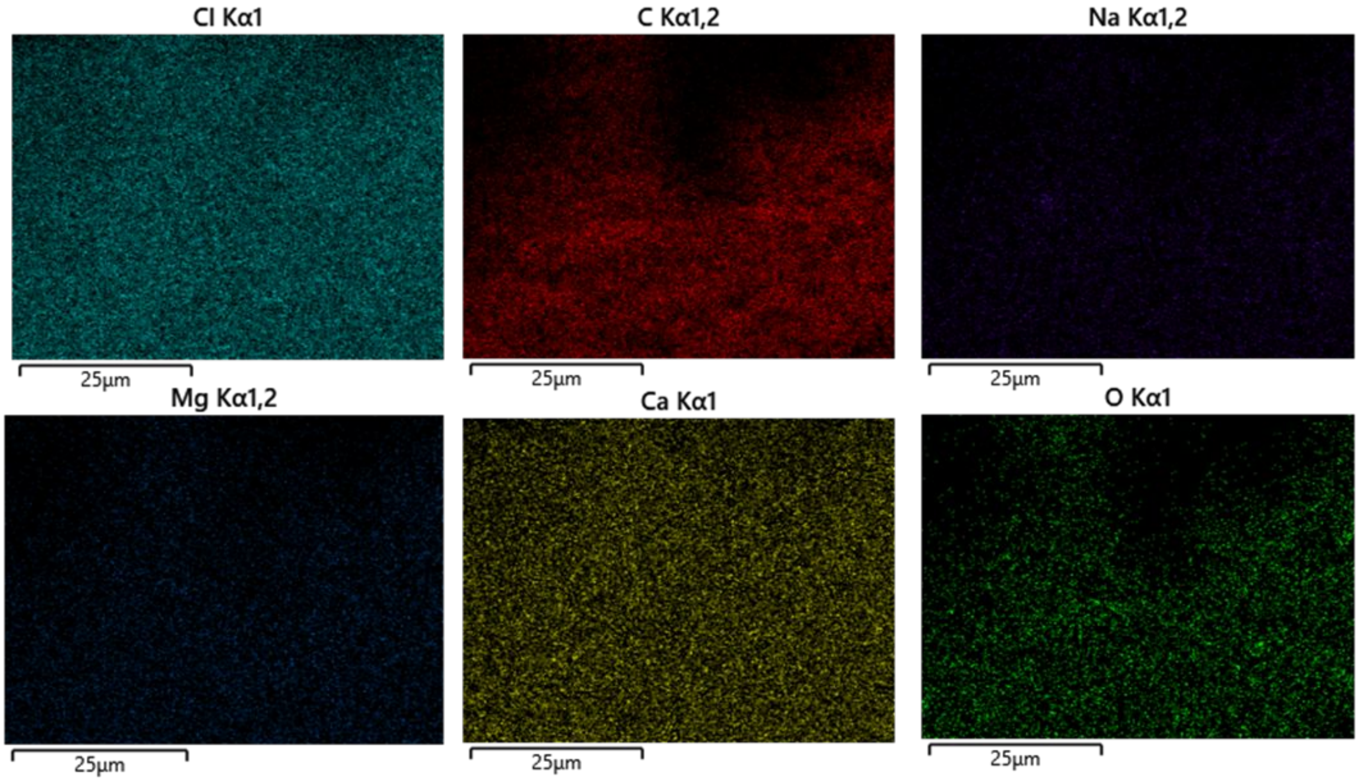

Element	Wt%
C	74.63
O	5.42
Na	0.52
Mg	0.62
Cl	12.89
K	0.45
Ca	5.1
Sr	0.37

Figure S3 – EDS images and data of activated PetCoke after contacting with Montney Brine

Table S1 – Zeta potential measurement; “ACT PT + Duvernay” and “ACT PT + Montney” are the activated PetCoke samples post water treatment for Duvernay FPW and Montney FPW respectively.

Simulated Brine (Montney)	Average Zeta Potential (mV)	Simulated Brine (Duvernay)	Average Zeta Potential (mV)
**ACT PT + Montney**	2.82	**ACT PT + Duvernay**	1.25
**ACT PT**	-6.71	**ACT PT**	-7.60
**PetCoke**	-2.94	**PetCoke**	-1.27

Table S2 – Trace metal analysis in FPW before and after activated carbon treatment

**Trace metals (μg L^{-1})**	**P**	**Sn**	**Tl**	**As**	**Se**	**Mo**	**Sb**	**Zn**	**Pb**	**Cd**	**Co**	**Ni**	**B**	**Si**	**Mn**	**Fe**	**Cr**	**V**	**Al**	**Be**	**Cu**	**Ag**	**Ti**	**Ce**	**Sr**	**Ba**	**Li**
Duvernay	< 96	< 116	< 112	< 140	< 156	< 100	< 280	1759	< 116	< 44	< 56	< 20	84615	27730	5841	23954	< 116	< 104	323	< 20	< 52	< 108	< 36	< 884	1231480	20671	63110
ACT PT treated Duvernay	175	< 116	< 112	< 140	< 156	166	< 280	714	< 116	< 44	< 56	893	64167	12878	5803	90	< 116	< 104	451	< 20	52	< 108	< 36	< 884	1253610	21007	70974
AC1 treated Duvernay	107	< 116	< 112	< 140	169	< 100	< 280	886	< 116	< 44	< 56	< 20	79277	22323	5855	< 84	< 116	< 104	322	< 20	64	< 108	< 36	< 884	1218220	21580	72550
AC2 treated Duvernay	< 240	< 290	< 280	< 350	< 390	< 250	< 700	653	< 290	< 110	< 140	< 50	77068	15647	5653	< 210	< 290	< 260	897	< 50	176	< 270	< 90	< 2210	1342100	21928	67011
AC3 treated Duvernay	< 240	< 290	< 280	< 350	< 390	< 250	< 700	379	< 290	< 110	< 140	< 50	84947	19022	5753	< 210	< 290	< 260	802	< 50	184	< 270	< 90	< 2210	1304190	22081	68448
Montney	7504	< 116	< 112	< 140	< 156	< 100	< 280	1434	< 116	< 44	< 56	< 20	28005	17046	6046	28356	< 116	< 104	332	< 20	< 52	< 108	< 36	< 884	1265240	12740	77148
ACT PT treated Montney	705	< 116	< 112	< 140	< 156	189	< 280	84	< 116	< 44	< 56	838	20475	8953	6104	< 84	< 116	< 104	279	< 20	58	< 108	< 36	< 884	1228410	9923	86553
AC1 treated Montney	5014	< 116	< 112	< 140	163	< 100	< 280	952	< 116	< 44	< 56	24	24988	20496	5873	31203	< 116	< 104	377	< 20	85	< 108	< 36	< 884	1293620	12040	84586
AC2 treated Montney	1924	< 290	< 280	< 350	< 390	< 250	< 700	1296	< 290	< 110	< 140	< 50	25758	49004	5814	20132	< 290	< 260	688	< 50	160	< 270	< 90	< 2210	1341850	14678	84119
AC3 treated Montney	1396	< 290	< 280	< 350	< 390	< 250	< 700	818	< 290	< 110	< 140	< 50	31166	15656	5708	13475	< 290	< 260	828	< 50	147	< 270	< 90	< 2210	1266970	13510	85558
